# Solution structure of the parvulin-type PPIase domain of *Staphylococcus aureus *PrsA – Implications for the catalytic mechanism of parvulins

**DOI:** 10.1186/1472-6807-9-17

**Published:** 2009-03-24

**Authors:** Outi Heikkinen, Raili Seppala, Helena Tossavainen, Sami Heikkinen, Harri Koskela, Perttu Permi, Ilkka Kilpeläinen

**Affiliations:** 1 Laboratory of Organic Chemistry, Department of Chemistry, P.O. Box 55, FI-00014 University of Helsinki, Finland; 2 Program in Structural Biology and Biophysics, Institute of Biotechnology, P.O. Box 65, FI-00014 University of Helsinki, Finland; 3 Finnish Institute for Verification of the Chemical Weapons Convention, P.O. Box 55, FI-00014 University of Helsinki, Finland

## Abstract

**Background:**

*Staphylococcus aureus *is a Gram-positive pathogenic bacterium causing many kinds of infections from mild respiratory tract infections to life-threatening states as sepsis. Recent emergence of *S. aureus *strains resistant to numerous antibiotics has created a need for new antimicrobial agents and novel drug targets. *S. aureus *PrsA is a membrane associated extra-cytoplasmic lipoprotein which contains a parvulin-type peptidyl-prolyl *cis-trans *isomerase domain. PrsA is known to act as an essential folding factor for secreted proteins in Gram-positive bacteria and thus it is a potential target for antimicrobial drugs against *S. aureus*.

**Results:**

We have solved a high-resolution solution structure of the parvulin-type peptidyl-prolyl *cis-trans *isomerase domain of *S. aureus *PrsA (PrsA-PPIase). The results of substrate peptide titrations pinpoint the active site and demonstrate the substrate preference of the enzyme. With detailed NMR spectroscopic investigation of the orientation and tautomeric state of the active site histidines we are able to give further insight into the structure of the catalytic site. NMR relaxation analysis gives information on the dynamic behaviour of PrsA-PPIase.

**Conclusion:**

Detailed structural description of the *S. aureus *PrsA-PPIase lays the foundation for structure-based design of enzyme inhibitors. The structure resembles hPin1-type parvulins both structurally and regarding substrate preference. Even though a wealth of structural data is available on parvulins, the catalytic mechanism has yet to be resolved. The structure of *S. aureus *PrsA-PPIase and our findings on the role of the conserved active site histidines help in designing further experiments to solve the detailed catalytic mechanism.

## Background

*Staphylococcus aureus *is a Gram-positive bacterium causing many kinds of infections from mild respiratory tract infections to life-threatening states as sepsis. It produces many toxins and has a remarkable ability to acquire resistance to antimicrobial drugs. Many *S. aureus *strains have acquired resistance to commonly used antibiotics and some strains are becoming multi-resistant. Methicillin-resistant strain of *Staphylococcus aureus *(MRSA) is the principal cause of severe nosocomial infections which can be fatal to compromised patients. Whole genome sequencing of two MRSA strains in 2001 was regarded as a way to find targets for novel antibiotics against infections caused by MRSA [1].

PrsA protein is found ubiquitously in Gram-positive bacteria, including *S. aureus *[Swiss-Prot:P60747], but not in Gram-negative ones [2,3]. By sequence homology PrsA contains a parvulin-type peptidyl-prolyl *cis-trans *isomerase (PPIase) domain and flanking N- and C-terminal domains. PPIases are enzymes that catalyze *cis-trans*-isomerization of the peptide bonds preceding proline residues [4]. Biological role of PPIases is to act as chaperones or foldases in protein folding and remodelling. FK506 binding proteins (FKBPs), cyclophilins and parvulins form the three classes of PPIases each having their own fold, substrate specificity and catalytic mechanism.

PrsA is localized at the space between plasma membrane and cell wall and it is bound to the cell membrane through a lipid-anchor attached to the N-terminal cysteine residue [2,3]. It has been shown to have a role as folding catalyst of secreted proteins. In bacteria, secreted proteins include enzymes important for formation of the cell wall and toxins. Due to importance of the catalyzed reaction in protein folding PrsA is a potential target for novel antimicrobial drugs. PrsA has been previously shown to be an essential protein for viability of *B. subtilis *[2].

Parvulin-type PPIases are ~100 residues long globular protein domains folding into a four-stranded antiparallel β-sheet core surrounded by four α-helices (βα3βαβ2 parvulin-fold) [4]. Parvulin-type PPIases have been found both in bacteria and in eukaryotes. At present there are structures of 7 different parvulins available in the Protein Data Bank: human Pin1 (e.g. [PDB:1PIN, 1NMV and 1NMW]) [5,6] and Par14 [PDB:1EQ3][7], Pin1At from *Arabidopsis thaliana *[PDB:1J6Y][8], Par10 [PDB:1JNS] [9] and SurA [PDB:1M5Y][10] from *Escherichia coli*, Ess1 from *Candida albicans *[PDB:1YW5][11] and PrsA-PPIase from *Bacillus subtilis *[PDB:1ZK6][12]. Also several other parvulin-type PPIases are known, e.g. Par27 from *Bordetella pertussis *[13], but their structures are still to be solved. The subtypes of parvulins differ in length and composition of the S_1_-H_1 _loop. In hPin1-type parvulins the loop has a high number of positively charged residues and this is thought to induce the preference for substrates having a negatively charged residue, preferably a phosphorylated serine/threonine, before the processed proline [5]. In Par14-type parvulins this loop is missing and in SurA PPIase domain I the S_1_-H_1 _loop contains mainly hydrophobic residues [7,10].

PrsA of *S. aureus *shows 24% amino acid sequence conservation to PrsA protein from *Bacillus subtilis *[Swiss-Prot:P24327]. The PPIase domain is the most conserved area of the sequence (42% of the residues conserved). Sequence comparison of *B. subtilis *and *S. aureus *PrsA-PPIases shows that they differ in length and nature of the S_1_-H_1 _loop. *S. aureus *PrsA-PPIase contains a long loop rich of lysine residues whereas in *B. subtilis *PrsA the loop is very short. This suggests that the structure and the substrate specificity of *S. aureus *PrsA-PPIase would rather resemble hPin1-type parvulins than *B. subtilis *PrsA-PPIase.

Since PrsA is known to be an essential protein for other gram-positive bacteria [2] it is a potential target for antimicrobial drugs against *S. aureus *infections. Exact knowledge of the structure and catalysis mechanism of PrsA-PPIase is a prerequisite for successful design of efficient and selective enzyme inhibitors to be used as antibacterial agents against Gram-positive bacteria. We have studied structure and function of the parvulin-type PrsA-PPIase from *S. aureus *using NMR spectroscopy.

## Results

### Protease-coupled PPIase assay

**Figure 1:**
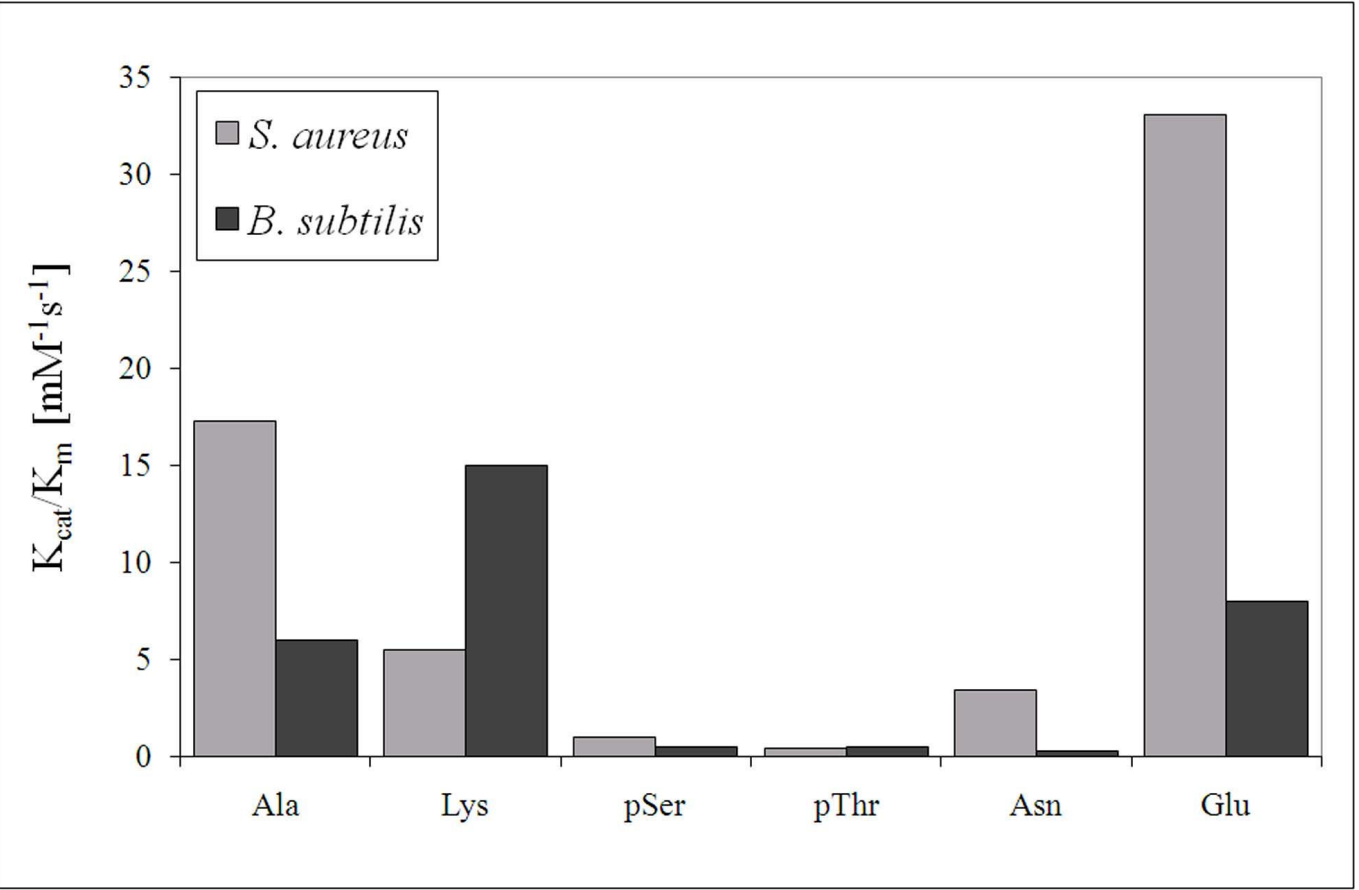
**Protease-coupled PPIase assay**. Catalytic activity of *S. aureus *and *B. subtilis *PrsAs towards Suc-AXPF-*p*NA tetrapeptides, where X = A, K, pS, pT, N or E.

The results of protease-coupled PPIase assay confirm the prolyl isomerase activity of *S. aureus *PrsA-PPIase (Figure 1). The highest catalytic activity (k_cat_/K_m _= 33 mM^-1^s^-1^) was observed with Suc-AEPF-*p*NA peptide. Comparison with the results obtained with PrsA-PPIase from *B. subtilis *indicates difference in the substrate preference. The enzyme from *S. aureus *prefers a substrate having a negatively charged residue before the proline residue (preferred substrate Suc-AEPF-*p*NA). The same enzyme from *B. subtilis *has the highest catalytic activity towards Suc-AKPF-*p*NA peptide.

### Structure determination

**Figure 2:**
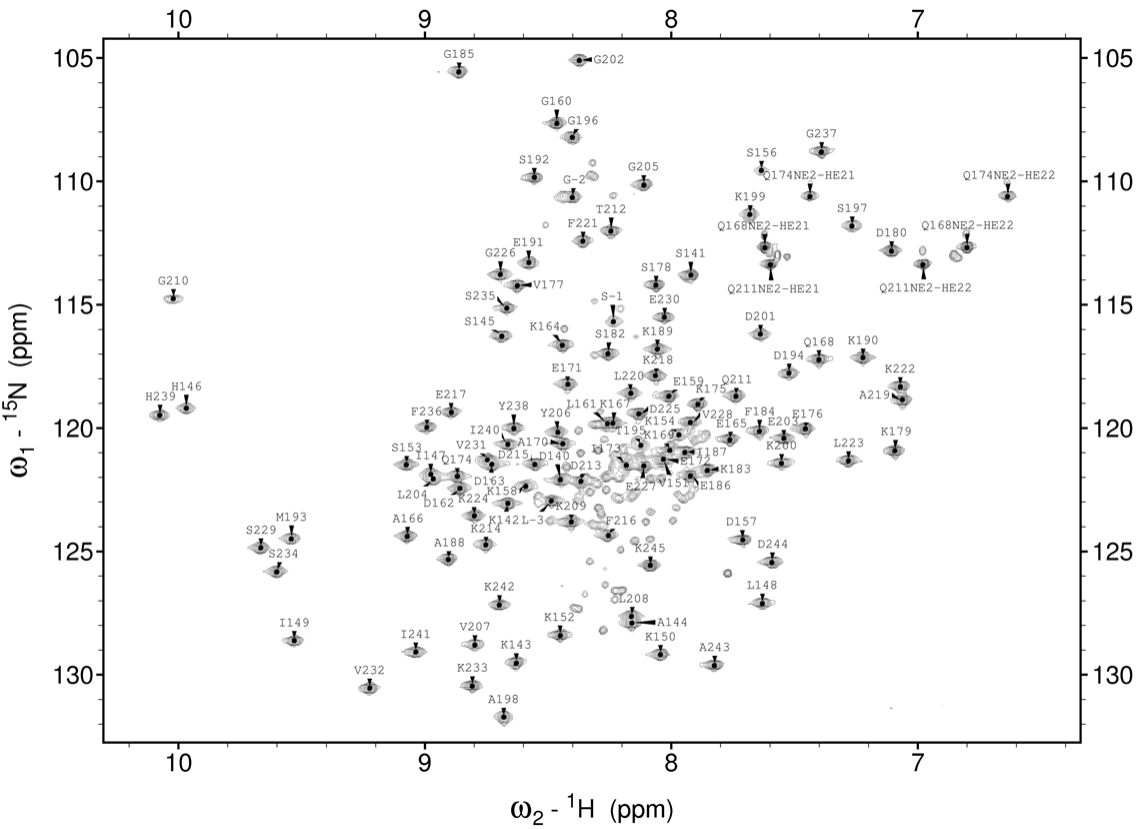
**^1^H-^15^N-HSQC spectrum of uniformly ^13^C^15^N-labeled *S. aureus *PrsA-PPIase (residues 140–245)**. The spectrum was recorded at 800 MHz field at 25°C. The protein concentration was ~1 mM and sample buffer was 20 mM Bis-Tris, pH 6.8. Resonance assignments of backbone and side chain amides are shown on each peak.

^1^H-^15^N-HSQC spectrum of PrsA-PPIase (Figure 2) shows clearly resolved signals of a well-folded protein. However, there is a second set of signals with about one fifth of intensity of the main signals. Despite attempts to change the sample conditions and the protein construct we were unable to remove this extra set of signals from the ^1^H-^15^N-HSQC spectrum. Both SDS-PAGE analysis and mass spectra indicate that there is only one kind of polypeptide present in the sample. Narrow distribution of the ^1^H chemical shifts of the minor signals implies that they originate from an unstructured polypeptide. A closer inspection of the NOESY spectra showed that these resonances have practically no NOEs. From this we concluded that the extra signals belong to an unfolded form of PrsA-PPIase which probably would not interfere with the structure determination and we proceeded with this sample. Sequential assignment of the extra signals (data not shown) indeed confirmed they originate from the same polypeptide sequence as the main signals.

All backbone amide signals except K155 are visible in the ^1^H-^15^N-HSQC spectrum and were sequentially assigned. Assignments were found for 97% of all ^1^H-, ^13^C- and ^15^N-resonances. Majority of the missing assignments belong to the overlapping side chain resonances of numerous lysine residues. Peak picking of the three-dimensional ^13^C- and ^15^N-edited NOESY-HSQC spectra yielded 2621 and 1242 cross-signals, respectively. Total of 2161 distance restraints were extracted from the NOESY spectra with the automatic NOESY signal assignment and torsion angle dynamics procedure of CYANA 2.1 software [14]. After the final molecular dynamics refinement with AMBER program [15] 25 structures were chosen to the representative structure family. The final set of structures contains neither distance restraint violations over 0.2 Å nor dihedral angle restraint violations exceeding 7°. According to the structure statistics (Table 1) and the quality analysis with PROCHECK-NMR [16] and WHAT_CHECK [17] the structure determination yielded a structure family of excellent quality. RMSD between the structures (residues 140–243) is 0.5 Å for the backbone and 1.0 Å for all the heavy atoms. If the somewhat less ordered residues of the S_1_-H_1 _loop (residues 153–159) are excluded, RMSD drops to 0.3 Å and 0.8 Å for the backbone and heavy atoms, respectively. Over 99% of the residues reside on the favoured regions of the Ramachandran plot.

**Table 1:** Structure statistics of PrsA-PPIase

**Total distance restraints**	2161
Short-range |i - j| ≤ 1	1081
Medium-range, 1 < |i - j| < 5	379
Long-range, |i - j| ≥ 5	764
Restraints per residue	19.5
**Violation statistics**	
Maximum NOE restraint violation (Å)	0.16
Number of NOE violations > 0.10 Å	3 ± 2
**Energies**	
Average restraint violation energy (kcal/mol ± SD)	9.55 ± 0.86
Average AMBER energy (kcal/mol ± SD)	-3259.69 ± 8.55
**RMS deviations from ideal covalent geometry**	
Bond lengths (Å ± SD)	0.0096 ± 0.0001
Bond angles (° ± SD)	1.93 ± 0.02
**Atomic coordinate RMSD (Å ± SD) for residues 140–243 and (140–152, 160–243)**	
Backbone atoms	0.55 ± 0.18 (0.31 ± 0.05)
Heavy atoms	1.07 ± 0.20 (0.80 ± 0.06)
**Ramachandran map regions (%)**	
Residues in most favoured regions	93.9
Additionally allowed regions	5.9
Generously allowed regions	0.2
Disallowed regions	0.0

**Figure 3:**
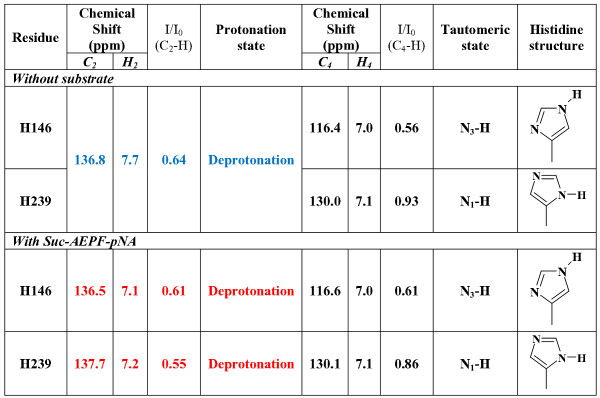
**Determination of the tautomeric state of the active site histidines**. See reference [18] for further details on interpretation of the results. Blue: Due to partially overlapping H_2_-C_2 _signals the protonation state determination yielded only an average value of the two peaks. The value suggests that the histidines are in deprotonated state. The Suc-AEPF-*p*NA titration however separated the two H_2_-C_2 _signals and enabled separate determination of the protonation states. Red: Unique assignment is not available, but the protonation state determination gives equivalent results for both peaks.

Since there is a distracting difference in the orientation of the two conserved active site histidines between the crystal [5,10,11] and the solution [6-9,12] structures of parvulin PPIases, we decided to look into the construction of the active site of the enzyme more closely. The results of protonation and tautomeric state determination of the active site histidines (see ref. [18] for interpretation of the results) are represented in Figure 3. Due to partially overlapping H146 and H239 H_2_-C_2 _signals the protonation state determination yielded only an average value of the two residues for peptide-unbound form of PrsA-PPIase. Based on the average value one can judge that the histidines are in deprotonated state. The Suc-AEPF-*p*NA titration however separated the two H_2_-C_2 _signals and enabled separate determination of the protonation states. Protonation state and tautomeric state of the active site histidines were not affected by the presence of substrate peptide Suc-AEPF-*p*NA. Both H146 and H239 are in deprotonated state but they differ in tautomeric state of the side chain. H146 is in N_3_-protonated state whereas H239 binds the proton through N_1 _(see Figure 3). Also the C_4 _chemical shifts of the histidines support this conclusion [19].

### Structure description

**Figure 4:**
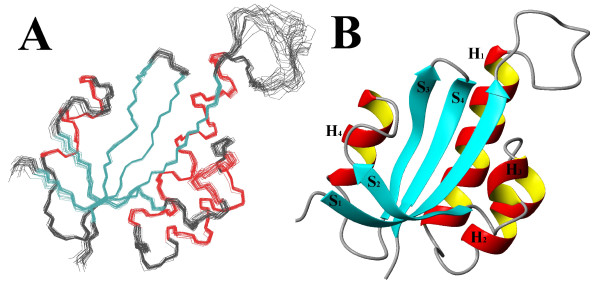
**Solution structure of *S. aureus *PrsA-PPIase**. The protein construct used in the study comprised residues 140–245 of the *S. aureus *PrsA [Swiss-Prot: P60747]. **(A) **Superimposed backbone traces of the 25 structures in the structure ensemble. Secondary structure elements are colour coded as: red – helix; cyan – strand; grey – coil. **(B) **A ribbon model of the average structure showing the codes of the secondary structure elements. Structure visualisation was done using MOLMOL [33].

**Figure 5:**
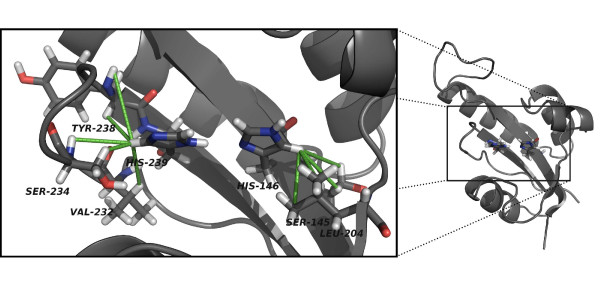
**Detailed structure of the *S. aureus *PrsA-PPIase active site histidines**. The most important NOE distance constraints determining the orientation of the active site histidine ring planes are indicated with green lines. This conformation of the histidines enables hydrogen bonding between the N_1 _positions of the histidine side chains. Visualisation was created using PyMol [34].

Structure of PrsA-PPIase (Figure 4) is a typical parvulin-fold consisting of a four-stranded antiparallel β-sheet core (S_1_, S_2_, S_3 _and S_4_) and four α-helices (H_1_, H_2_, H_3 _and H_4_) surrounding it. PrsA-PPIase has a ten-residue extended loop containing four positively charged lysine residues (K152, K154, K155 and K158) between S_1 _and H_1_. This loop is more loosely defined compared with the rest of the structure, and it lacks regular secondary structure. The hydrophobic core of the protein on the concave side of the β-sheet is formed by L204, V207, F216, L220 and I241 and by the two histidines (H146 and H239) occupying the active site. On the convex side of the β-sheet the main hydrophobic residues attaching the helices H_1 _and H_2 _are I147, V177, F184, V228 and I240. The antiparallel orientation of the active site histidine ring planes brings the N_1 _nitrogens next to each other (Figure 5). The differing tautomeric state of the two histidines enables a hydrogen bond to be formed between the histidine rings.

### Peptide titrations

**Figure 6:**
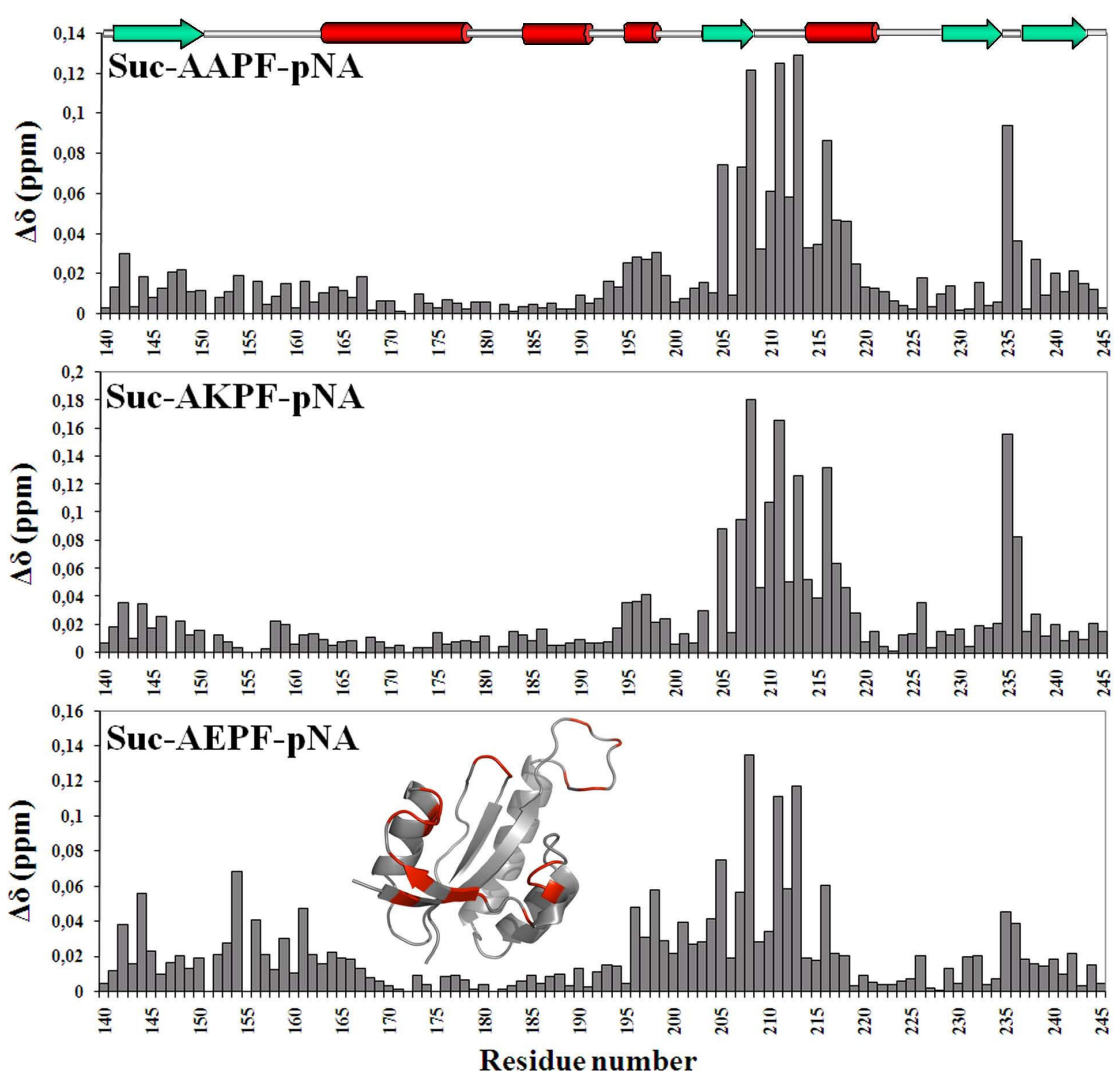
**Chemical shift mapping of PrsA-PPIase during Suc-AXPF-*p*NA titrations**. Chemical shift perturbations induced to the backbone amide signals of *S. aureus *PrsA-PPIase upon Suc-AXPF-*p*NA (X = A, K or E) peptide titration. Position of the secondary structure elements in the sequence is shown at the top of the graph: green arrow – β-strand, red bar – α-helix. Structure insert shows the location of residues which evinced the largest chemical shift perturbations in the Suc-AEPF-*p*NA titration (red – Δδ > 0.03 ppm).

Location of the active site and structural changes during enzyme action were probed by titration experiments with proline containing tetrapeptides previously shown to be substrates for parvulin PPIases [20]. Titration of PrsA-PPIase with Suc-AXPF-*p*NA (X = A, K or E) substrate peptides induced chemical shift perturbations in the ^1^H-^15^N-HSQC spectrum. However, large excess of peptide was needed in all three cases in order to achieve clear changes in the spectrum. The binding affinity of the peptides was in millimolar range (data not shown). Largest chemical shift perturbations were observed at helix H_3_, strand S_2 _and at S_2_-H_4 _and S_3_-S_4 _loops which reflects the typical substrate binding site of parvulins (Figure 6) [5]. Upon titration with the Suc-AEPF-*p*NA peptide chemical shift changes were observed also at the S_1_-H_1 _loop. This behaviour was not observed with other peptides used. The S_1_-H_1 _loop contains a cluster of positively charged lysines which presumably participate in binding of the negatively charged glutamate side chain of the substrate. During Suc-AEPF-*p*NA titration chemical shift changes of the aromatic side chains were also followed using ^1^H-^13^C-HSQC spectrum. H_2 _proton resonances of the both active site histidines moved about 0.5 ppm upfield (see Figure 3) which reflects a close proximity to the peptide binding site.

### Dynamics and exchange

**Figure 7:**
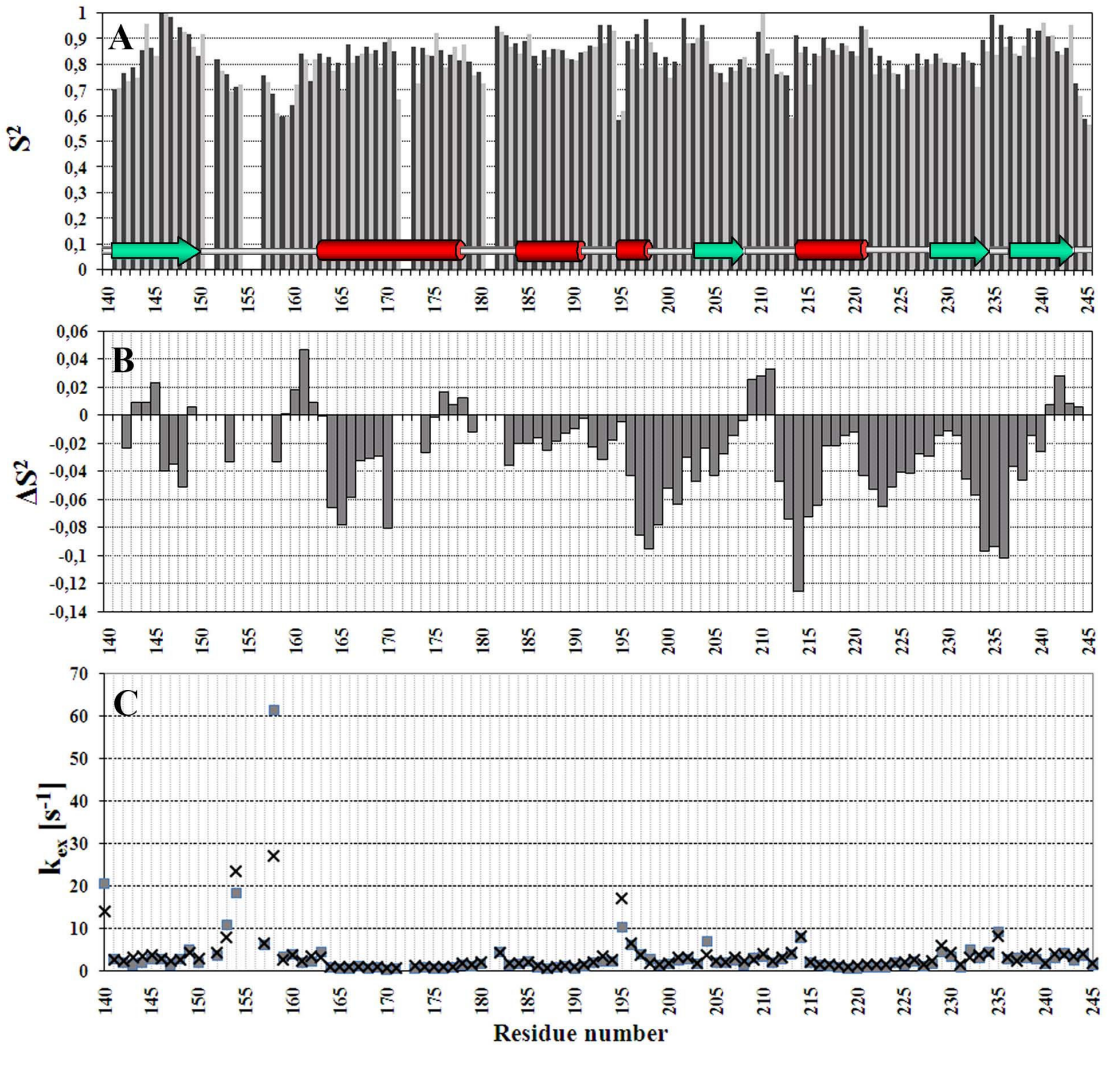
**Dynamical features of *S. aureus *PrsA-PPIase**. Generalized order parameters (S^2^) for backbone amides were calculated using ModelFree formalism from ^15^N R_1 _and R_2 _relaxation rates and ^1^H-^15^N heteronuclear NOEs. **(A) **Generalized order parameter (S^2^) as function of residue sequence for PrsA-PPIase (dark grey) and for PrsA-PPIase + Suc-AEPF-*p*NA (light grey). **(B) **S^2 ^changes induced upon Suc-AEPF-*p*NA peptide addition averaged over three consecutive residues. **(C) **Backbone amide proton exchange rates as function of residue sequence for PrsA-PPIase (box) and PrsA-PPIase + Suc-AEPF-*p*NA (cross). Position of the secondary structure elements in the sequence is shown on the top panel: green arrow – β-strand; red bar – α-helix.

The dynamical behaviour of the protein was depicted through generalized order parameter S^2^, which was extracted from R_1 _and R_2 _relaxation rates and heteronuclear NOEs of the backbone amides. Additional information on dynamics was gained through backbone amide exchange rates. The generalized order parameters and the exchange rates were determined both in presence and in absence of the Suc-AEPF-*p*NA peptide substrate. The data for K155 and S156 and the relaxation data for D140 are missing due to low intensity of the ^1^H-^15^N-HSQC signals, and the data for V151 and E172 are missing due to signal overlap. The relaxation analysis was successfully applied for all the residues containing complete set of relaxation data. Graph of generalized order parameter S^2 ^as function of sequence (Figure 7a) shows an overall S^2 ^of about 0.8 and a consistent decrease at the S_1_-H_1 _loop. Presence of Suc-AEPF-*p*NA peptide induced changes in the order parameter at the loops surrounding the active site but also at the H_1 _helix (Figure 7b). The backbone amide proton exchange rates are slow within the secondary structure elements and the regions of faster exchange reflect the dynamical behaviour of the protein demonstrated by the order parameters (Figure 7c). The fastest exchange rates are observed for residues at the S_1_-H_1 _loop and at the loops facing the active site cavity.

## Discussion

In this study we have investigated the structure and function of the parvulin-type PPIase domain of PrsA protein from *S. aureus*. NMR spectroscopic structure determination of PrsA-PPIase yielded a high-quality structure which enabled investigation of the catalytic site in detail. Solution structure of PrsA-PPIase shows close structural similarity to hPin1-type parvulins but also some important differences in constitution of the active site. The original hypothesis on the catalysis mechanism of the parvulin-type PPIases is based on the crystal structure of hPin1 [5]. However, the recent studies of hPin1 [21-23] have provided new insight into the functional status of the active site residues and thus have brought the original catalysis mechanism into question. The solution structure of *S. aureus *PrsA-PPIase supports these findings but also brings out some new aspects into the debate.

The results of protease-coupled PPIase assay indeed confirm that PrsA-PPIase functions as a prolyl-isomerase. The most efficient catalysis was observed with Suc-AEPF-*p*NA peptide. The substrate preference of *S. aureus *PrsA-PPIase resembles that of hPin1 which was somewhat expected based on common S_1_-H_1 _loop rich of positively charged residues. Binding of multivalent anions to the S_1_-H_1 _loop of PrsA-PPIase was also confirmed by NMR titrations with sodium sulphate (data not shown). Clear chemical shift perturbations resembling the ones Bayer *et al. *observed with hPin1 [6] were detected at the S_1_-H_1 _loop. Paradoxically, the protease-coupled PPIase assay showed practically no prolyl-isomerase activity towards Suc-A(pS/pT)PF-*p*NA peptides.

**Figure 8:**
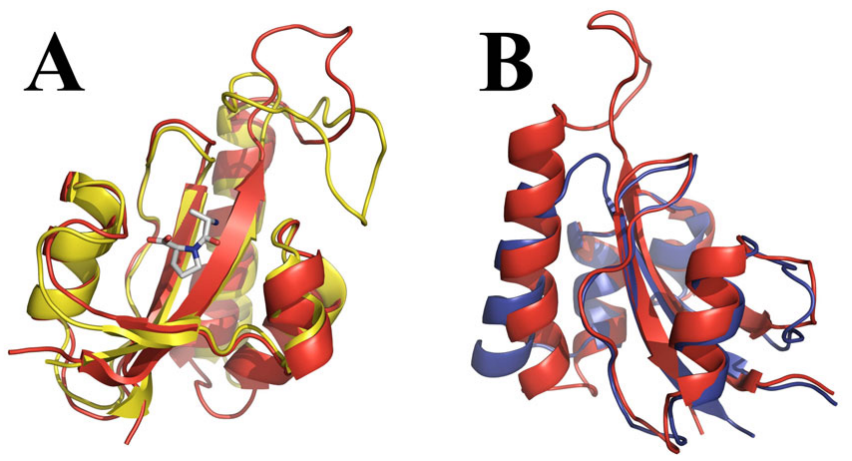
**Comparison of *S. aureus *PrsA-PPIase structure to hPin1 and *B. subtilis *PrsA**. Superimposition of the *S. aureus *PrsA-PPIase (red) **(A) **with the crystal structure of hPin1 complexed with AlaPro dipeptide (yellow) [PDB:1PIN] [5] and **(B) **with the solution structure of PrsA-PPIase from *B. subtilis *(blue) [PDB:1ZK6][12]. Note that the two structure overlays are presented from different perspectives to highlight the differences of the two structures. Structure superimpositions and visualisations were done using PyMol [34].

The overall structure of PrsA-PPIase shows resemblance to the previously published parvulin PPIase structures. A structure similarity search with DALI program [24] gave hPin1 [PDB:1F8A] and *C. albicans *Ess1 [PDB:1YW5] as the two closest hits with 1.7 and 2.0 Å backbone RMSDs, respectively. The H_1_-S_1 _loop of positively charged residues is also present in hPin1 and Ess1. Being a potential target for anticancer drugs, the human mitotic regulator hPin1 is the most studied parvulin. Overlay of PrsA-PPIase and the crystal structure of hPin1 containing AlaPro dipeptide substrate [5] shown in Figure 8a indicates that the largest differences between the two structures are found at the loosely defined S_1_-H_1 _loop and at the H_1_-H_2 _loop. PrsA-PPIase of *B. subtilis *lacks the extended loop between the S_1 _sheet and the H_1 _helix. Overlay of the secondary structure elements the two PrsA-PPIases reveals the difference in the orientation and length of the H_1 _helix (Figure 8b). Otherwise the backbone traces of the two PrsA-PPIases are fairly similar.

The active site of PrsA-PPIase was mapped by NMR titrations with parvulin substrate peptides. Our results conform well with the previous studies with other parvulins [5,12]. Largest chemical shift changes occurred at H_3 _helix, S_2 _strand and at S_2_-H_4 _and S_3_-S_4 _loops which face the active site and contain the residues thought to participate in the catalysis mechanism (Figure 6). Based on the NMR titrations the dissociation constant for all tested peptides was in millimolar regime and most of the spectral changes were practically the same with all the three peptides. During the Suc-AEPF-*p*NA peptide titration, but not with the other peptides, we observed consistent chemical shift perturbations at the S_1_-H_1 _loop. Backbone amide titration data demonstrates involvement of the S_1_-H_1 _loop in substrate binding when the substrate contains a negatively charged glutamate residue before the processed proline.

Referring to previously published NMR titration data, Bailey *et al. *concluded recently that parvulin active site histidines are not involved in substrate binding [23]. It should be noted, however, that NMR chemical shift perturbation studies are commonly done using only backbone N-H correlations (i.e. using ^1^H-^15^N-HSQC spectrum). Participation of the active site histidines (H146 and H239) in the substrate binding is not easily observed in ^1^H-^15^N-HSQC-based NMR titrations since backbone amides reside quite far from the peptide binding site. Involvement of the histidine side chains in substrate binding is however clearly evidenced by the chemical shift perturbations in the ^1^H-^13^C-HSQC spectrum of the aromatic residues (see Figure 3).

**Figure 9:**
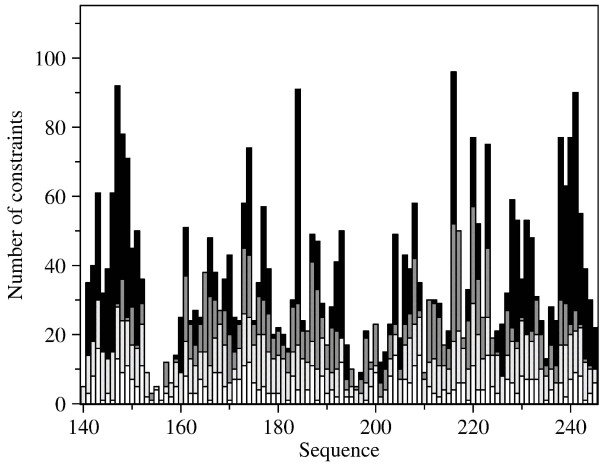
**Sequential distribution of the NOE distance constraints used in the structure calculation**. The NOE distance constraints have been classified according to their range (R) as: Black – long-range (R ≥ 5); dark grey – medium-range (5 > R > 1); grey – sequential (R = 1); white – intraresidual (R = 0). Graph was created with CYANA 2.1 [14].

A plot of generalized order parameters S^2 ^as function of sequence demonstrates a tightly folded protein (Figure 7a). Reduced S^2 ^values at the S_1_-H_1 _loop indicate that the loop is more flexible than the rest of the structure. Conformational variation of the structure family at the S_1_-H_1 _loop is thus an indication of real dynamic behaviour, not solely a lack of NOE distance restraints (Figure 9). This flexibility enables induced fit mechanism during binding of negatively charged substrate peptides. Especially interesting is the rather low S^2 ^of T195. This threonine also stands out in the amide proton exchange rate plot with a high exchange rate (Figure 7c). Order parameter of the solvent exposed T195 might be distorted due to chemical exchange of the amide proton with water. Presence of peptide substrate (Suc-AEPF-*p*NA) induces some changes in the order parameters and exchange rates (Figure 7b–c). Most of the changes are, as expected, at the loops surrounding the active site and carrying the catalytic residues. However, somewhat unexpected changes are observed in the helix H_1 _which is rather distant from the peptide binding site. Since the relaxation experiments were conducted in presence of Suc-AEPF-*p*NA peptide, which has a negatively charged residue before the proline, the changes in S^2 ^values for the H_1 _helix might be due to rearrangement of the S_1_-H_1 _loop during peptide binding. The S_1_-H_1 _loop can be acting as a hinge twisting the H_1 _helix.

**Figure 10:**
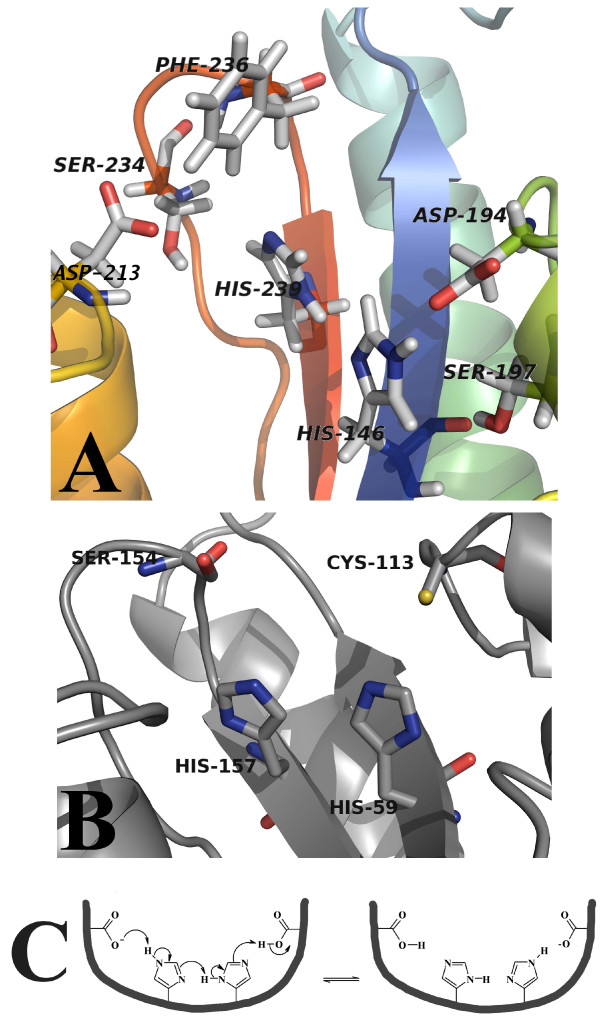
**The differences between the active site residues of parvulins**. Comparison of PrsA-PPIase and hPin1 active site residues shows that the highly conserved histidine side-chains have distinct conformations. **(A) **The active site residues of *S. aureus *PrsA-PPIase. **(B) **The hPin1 residues [PDB:1PIN] proposed to participate in the catalysis mechanism. **(C) **Schematic presentation of the potential charge relay system of *S. aureus *PrsA-PPIase. Deprotonation of the active site aspartates can be promoted by delocalisation of the negative charge through the active site histidines. Protein structure visualisations were done using PyMol [34].

Tautomeric state of the active site histidine residues was determined using NMR spectroscopy (Figure 3). The active site histidines are highly conserved in all parvulin PPIases. There is a systematic discrepancy regarding the conformation of these histidines between the crystal structures and the solution structures [5-12]. In the crystal structures the histidine side chain N_1 _nitrogens point to opposite directions whereas in the solution structures they face each other (Figures 10a–b). The ring planes of both histidines are flipped 180° changing the exact location of the ring nitrogens. The NOE distance restraints define the orientation seen in the NMR structures unambiguously (Figure 5). This difference is crucial regarding the discussion on the catalysis mechanism. The original hypothesis of the catalysis mechanism of parvulin PPIases was made based on the crystal structure of hPin1 [5]. Changing the orientation of the histidine ring planes affects the location of the transferable protons of the imidazole moieties. Using NMR spectroscopy we were also able to determine the tautomeric state of the histidines: in *S. aureus *PrsA-PPIase H146 is N_3_-protonated and H239 is N_1_-protonated (Figure 3). This combination of tautomeric states enables hydrogen bonding between the N_1 _nitrogens of the two histidines and it also creates prerequisites for a charge relay system through the active site of the enzyme (Figure 10c) [25]. The role of the parvulin active site histidines was recently discussed in detail by Bailey *et al. *[23]. Using a thorough array of active site histidine mutants of hPin1 they showed that the histidines are not essential for the catalytic activity of hPin1 but rather have a structural role and impact on the stability of the PPIase domain. It was noted that the capability of these residues to form hydrogen bonds is not an absolute requirement for proper enzyme function and that the double-mutant H59L/H157L even exhibited surprisingly high activity. The double-mutant was regarded to enhance the integrity of the catalytic site and the stability of the enzyme through additional hydrophobic contacts in the protein core. The hydrogen bonding between the active site histidines might also be a way to stabilise the parvulin fold. When this interaction is interrupted by single histidine mutation the stabilisation is lost but the double-mutant, even through a different mechanism (i.e. hydrophobic contacts), restores some of the stabilisation. Whether the histidines actively participate in the catalysis or solely serve as structural support for the catalytic machinery remains to be confirmed.

The original parvulin catalysis mechanism presented by Ranganathan *et al. *[5] has been disproved [21-23]. Comparison of the active site structure of *S. aureus *PrsA-PPIase and other parvulin PPIases questions the residues proposed to be responsible for the catalysis in parvulins [5]. The cysteine residue C113 of hPin1 was originally claimed to act as a nucleophile starting the catalysis (Figure 10b). This residue is replaced in *S. aureus *and in *B. subtilis *PrsAs by an aspartate (D194 and D154, respectively) which is also a potential nucleophile. In fact, Behrsin *et al. *have proved that the Pin1 C113D mutant remains functional [21]. Forthcoming steps of the original catalysis mechanism [5] include participation of a serine residue (S154 of hPin1) acting as a proton donor. In *S. aureus *PrsA-PPIase this residue is replaced by phenylalanine (F236) which is not capable of carrying out protonation/deprotonation steps. The same situation is also faced with other parvulin PPIases e.g. *E. coli *Par10 [9] and hPar14 [7]. In some parvulin PPIases this residue is replaced by valine and for example in *B. subtilis *PrsA by tyrosine [12]. Evidently the original catalysis mechanism proposed based on the crystal structure of hPin1 cannot be a universal route of the reaction for all parvulins. A closer inspection of the active site of *S. aureus *PrsA-PPIase reveals a somewhat symmetric assembly of aspartate and serine residues on both sides of the histidine pair (Figure 10a). Similar set of residues is also found in *B. subtilis *PrsA [12]. Mutation studies of *B. subtilis *PrsA have shown that D154A substitution (corresponding to D194 in *S. aureus *PrsA) destroys only half of the catalytic activity of PrsA [2]. Obviously some other residue can perform the role of the nucleophilic residue when it is inactivated by mutation. The symmetrical assembly of aspartates and serines and the charge relay system through the active site histidines would imply a protonation/deprotonation step as part of the catalytic mechanism. The charge relay system (Figure 10c) could facilitate deprotonation of the aspartates which would enhance their nucleophilic character. In light of the diverse structural and functional data on parvulin PPIases one inevitably raises a question whether all parvulin PPIases even share the same catalysis mechanism.

## Conclusion

The solution structure of PrsA-PPIase from *S. aureus *enables detailed study of its function and target based design of inhibitors. Highly conserved protein sequences are also found in other *Staphylococcus *subspecies. Exact biological role and importance of PrsA are still unclear although it is known to act as a foldase of secreted proteins (e.g. bacterial toxins) [3] and it is shown to be essential for *B. subtilis *[2]. Natural substrates of *S. aureus *PrsA-PPIase are not known at present, but the enzyme may prefer substrates where a negatively charged residue precedes the processed proline. The structure of the catalytic site of *S. aureus *PrsA-PPIase conflicts with the original hypothetical catalysis mechanism of parvulin PPIases. Recent studies also recognize the deficiencies of the parvulin catalysis mechanism [21-23]. The orientation and the tautomeric state of the active site histidine residues of *S. aureus *PrsA-PPIase suggest that the catalytic mechanism includes a protonation/deprotonation step facilitated by a charge relay system through the active site histidine pair. On the other hand, the hydrogen bonding between the active site histidines might merely serve as a structural stabilisation mechanism of the enzyme fold. Apparently the catalysis mechanism of parvulin-type PPIases still needs some clarifications. Existing structural data on parvulins can be used to design further experiments, e.g. site-directed mutagenesis, to decipher the detailed catalysis mechanism.

## Methods

### Protein expression and purification

The PPIase domain (residues 140–245) was expressed as glutathione S-transferase (GST) fusion. The protein was overexpressed in *E. coli *BL21 strain containing the pGEX-2T expression vector (GE Healthcare). For enzymatic studies, the cells were grown and harvested as described earlier [2]. For NMR samples, the cells were grown in M9 medium containing either ^15^NH_4_Cl as the sole nitrogen source or [^13^C_6_]-D-glucose/^15^NH_4_Cl as the sole carbon and nitrogen sources, respectively. The expression of protein was induced by addition of 1 mM isopropyl-β-D-thiogalactopyranoside (IPTG) at A_600 _of 0.8. The cells were grown 4 additional hours and harvested. For both enzymatic and NMR studies the cells were broken by French Press and centrifuged. The supernatant was applied to a glutathione-Sepharose FF (GE Healthcare) column, and washed with phosphate-buffered saline (PBS). The precission protease was added to the column and incubated 4 h at +5°C to release the protein. The cleaved protein was eluted with PBS, and the fractions containing the protein were concentrated with Vivaspin 2 (Sartorius Stedim Biotech). For NMR samples, the buffer was changed to 20 mM Bis-Tris pH 6.8, and D_2_O was added to the final concentration of 8% (v/v). The final protein concentration was ~1 mM.

### Protease-coupled PPIase assay

Prolyl isomerase activity of PrsA-PPIase was determined with α-chymotrypsin-coupled PPIase assay as described by Fischer *et al. *[26]. The catalytic activity was tested with synthetic succinyl-AXPF-*p*-nitroanilide (Suc-AXPF-*p*NA) peptides where X is alanine (A), lysine (K), asparagine (N), glutamic acid (E), phosphoserine (pS) or phosphothreonine (pT). The peptides with A, K and E were purchased from Bachem (Bubendorf, Switzerland). Suc-ANPF-*p*NA was synthesized by Ale Närvänen in University of Kuopio, Finland. The phosphorylated peptides were purchased from EZBiolab Inc. (Westfield, IN). *p*-Nitroanilide was cleaved off by α-chymotrypsin and the increase of released *p*-nitroanilide was monitored in absorbance at 390 nm. Cyclophilin from calf thymus (Sigma-Aldrich) was used as a positive control.

### Structure determination

NMR spectroscopy for the structure determination was performed on Varian INOVA 600 MHz and 800 MHz spectrometers with 5 mm inverse z-gradient triple-resonance probe heads at 25°C. The acquisition and processing were conducted with VNMR 6.1C software (Varian Inc., Palo Alto, CA). A conventional set of three-dimensional triple resonance experiments i.e. iHNCA [27], HN(CO)CA, HNCACB, HN(CO)CACB, HNCO, HN(CA)CO [28,29] was recorded for sequential backbone assignment. The aliphatic side chain resonances were assigned using three-dimensional HCCH-COSY and HCCH-TOCSY experiments with the help of CC(CO)NH and HCC(CO)HN experiments [28]. (Hβ)Cβ(CγCδ)Hδ, (Hβ)Cβ(CγCδCε)Hε [30] experiments and ^13^C-edited three-dimensional HSQC-NOESY spectrum were used in assignment of aromatic side chain resonances. Sparky 3.110 program [31] was used to analyze the NMR spectra.

The distance restraints for structure calculation were extracted from signal intensities of ^15^N- and ^13^C-edited three-dimensional HSQC-NOESY spectra. Automated NOESY signal assignment and structure calculation was conducted with CYANA 2.1 software [14]. In addition to NOE derived distance restraints, 146 φ and ψ dihedral angle constraints (average of the TALOS database hits used in the prediction ± 2 SD) were generated from chemical shift data with TALOS program (version 2003.027.13.05) [32]. After torsion angle dynamics run 40 structures were chosen from 400 calculated structures based on lowest target function value. These 40 structures were refined with molecular dynamics using Born implicit solvent model in AMBER 8.0 [15]. The final ensemble of 25 structures was chosen based on lowest AMBER energy and restraint violation energy. Quality of the final structures was analyzed with PROCHECK-NMR [16] and WHAT_CHECK [17] programs. Tautomeric state of the active site histidines H146 and H239 was determined using J_CN _intensity modulated constant time ^1^H-^13^C-HSQC spectrum [18] both in presence and in absence of the substrate peptide Suc-AEPF-*p*NA. Molecule visualization programs MOLMOL [33] and PyMOL [34] were used in preparation of the figures representing the protein structure.

### Peptide titrations

The Suc-AXPF-*p*NA tetrapeptides, where X = A, K or E, were tested for binding to PrsA-PPIase. The ^1^H-^15^N-HSQC-based titration experiments were conducted with 0.3 mM ^15^N-labeled PrsA-PPIase samples adding the unlabeled peptide as concentrated solution in sample buffer. The ^1^H-^15^N-HSQC spectrum was recorded after each peptide addition. Large excess of peptide was used at the last titration point to obtain high proportion of ligand-bound form of the protein. Total chemical shift change of the backbone amide signals at the titration end-point was calculated with the equation Δδ = [(0.17* Δδ_N_)^2^+(Δδ_H_)^2^]^1/2^. Determination of ^15^N relaxation rates, heteronuclear NOEs, amide proton exchange rates and tautomeric state of the histidine side chains in presence of Suc-AEPF-*p*NA peptide substrate were done at the titration end point (20-fold excess of peptide to protein).

### Protein dynamics

^15^N R_1 _and R_2 _relaxation rates of the backbone amide groups were measured using three-dimensional relaxation rate-resolved ^1^H-^15^N-HSQC spectra [35,36]. Inverse Laplace transform was applied to the relaxation dimension enabling extraction of the relaxation rate constants simply by peak picking. Heteronuclear NOEs of the backbone amide nitrogens were determined with conventional methods [37]. The data for the analysis of protein dynamics was recorded with a Bruker DRX 500 MHz spectrometer equipped with a 5 mm z-gradient inverse broadband probehead from 0.3 mM ^15^N-labeled PrsA-PPIase sample. Generalized order parameters for each backbone amide were extracted from the relaxation data using Modelfree 4.1 program [38,39] with FASTModelfree interface [40]. Proton exchange rates between backbone amides and water were measured through exchange rate-resolved ^1^H-^15^N-HSQC spectrum [41]. Relaxation and exchange rates as well as heteronuclear NOEs were determined both in absence and in presence of substrate peptide Suc-AEPF-*p*NA.

### Data deposition

The resonance assignments of *S. aureus *PrsA-PPIase and the distance constraints used in structure calculation have been deposited in BioMagResBank under accession number 15628. The atomic coordinates of *S. aureus *PrsA-PPIase structure ensemble have been deposited in Protein Data Bank under accession code 2JZV.

## Authors' contributions

OH carried out the NMR structure determination, peptide titrations, analyzed the NMR relaxation data and wrote the manuscript. RS prepared all protein samples used in the study, carried out the protease-coupled PPIase assays, participated in the design of the study and wrote the manuscript. HT conducted peptide titrations. SH and HK recorded and processed the NMR relaxation and exchange data. PP recorded the NMR spectra for structure determination, participated in the design of the study and wrote the manuscript. IK participated in the design of the study and wrote the manuscript. All authors read and approved the final manuscript.
